# Selenides and Diselenides: A Review of Their Anticancer and Chemopreventive Activity

**DOI:** 10.3390/molecules23030628

**Published:** 2018-03-10

**Authors:** Mónica Álvarez-Pérez, Wesam Ali, Małgorzata Anna Marć, Jadwiga Handzlik, Enrique Domínguez-Álvarez

**Affiliations:** 1 Instituto de Química Orgánica General, Consejo Superior de Investigaciones Científicas (IQOG, CSIC), Juan de la Cierva 3, 28006 Madrid, Spain; monica.a.p@csic.es; 2 Division of Bioorganic Chemistry, School of Pharmacy, Saarland University, Campus B2 1, D-66123 Saarbruecken, Germany; s8wealii@stud.uni-saarland.de; 3 Department of Technology and Biotechnology of Drugs, Faculty of Pharmacy, Jagiellonian University Medical College, Medyczna 9, 30-688 Cracow, Poland; marcmalgorzata@gmail.com (M.A.M.); j.handzlik@uj.edu.pl (J.H.)

**Keywords:** selenium, selenocompounds, selenides, diselenides, antioxidants, chemoprevention, anticancer compounds, cytotoxicity, cytostaticity

## Abstract

Selenium and selenocompounds have attracted the attention and the efforts of scientists worldwide due to their promising potential applications in cancer prevention and/or treatment. Different organic selenocompounds, with diverse functional groups that contain selenium, have been reported to exhibit anticancer and/or chemopreventive activity. Among them, selenocyanates, selenoureas, selenoesters, selenium-containing heterocycles, selenium nanoparticles, selenides and diselenides have been considered in the search for efficiency in prevention and treatment of cancer and other related diseases. In this review, we focus our attention on the potential applications of selenides and diselenides in cancer prevention and treatment that have been reported so far. The around 80 selenides and diselenides selected herein as representative compounds include promising antioxidant, prooxidant, redox-modulating, chemopreventive, anticancer, cytotoxic and radioprotective compounds, among other activities. The aim of this work is to highlight the possibilities that these novel organic selenocompounds can offer in an effort to contribute to inspire medicinal chemists in their search of new promising derivatives.

## 1. Introduction

**Figure 1:**
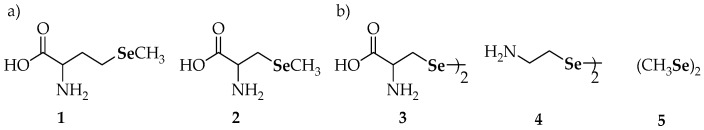
Naturally occurring selenides (**a**) and diselenides (**b**).

Various authors have extensively reviewed the different applications of selenium in human health [1,2,3,4]. Selenium is an essential element that forms part of the selenoaminoacids selenomethionine (**1**, Figure 1a) and selenocysteine (**2**). The latest is an essential constituent of the selenoproteins [5,6,7] and its deficiency can cause serious disorders [1,2]. Selenoproteins have crucial functions for human health. The most known are the gluthathione peroxidases, the iodothyronine deiodinases, the thioredoxin reductases and the selenoprotein P. The different glutathione peroxidases (GPx) are involved in the elimination of free radicals; the iodothyronine deiodinases enable the activation and deactivation of thyroid hormones. Thioredoxin reductases regenerate the thioredoxin (an important antioxidant class of proteins), and the selenoprotein P is involved in the transport of selenium in plasma and in the antioxidant defense against free radicals in lipids [5,6,7]. 

An intense research in the chemopreventive and/or anticancer activity of organic and inorganic selenium-containing compounds has been developed, as has been reviewed by different authors [8,9,10,11,12,13,14]. The duality of selenium as an antioxidant and as a prooxidant agent provides the basis for its potential applications in both cancer prevention and treatment. This dual chemopreventive and anticancer action has been extensively discussed or reported by different authors [15,16,17,18]. Regarding the chemopreventive activity, a mechanism usually exerted by different selenocompounds is the glutathione peroxidase-like activity [19]. Although inorganic selenocompounds may be superior chemopreventive agents than organic ones, current research is focused on the latter group due to their lower toxicity risk [20]. Among the inorganic selenocompounds, selenite can be highlighted by its chemopreventive [21] and anticancer action [22].

On the other hand, the mechanisms of action of the organic selenocompounds are very diverse [23]. Some of the most frequent are the reduction of oxidative stress through the elimination of free radicals [24,25], i.e., the induction of mutations [26], the cytotoxic activity [27] and the triggering of apoptotic events [28,29]. Nevertheless, certain selenocompounds can act through other less usual mechanisms, such as the inhibition of angiogenesis [30], the inhibition of the efflux pumps in cancer multidrug resistant cell lines [31] and the enhancement of the activity of chemotherapeutic drugs [32]. The chemical structure of anticancer and chemopreventive organic selenocompounds also shows a great variety, including selenocyanates [33], selenoureas [34], selenoesters [35], heterocycles with endocyclic selenium [36], selenium nanoparticles [37], selenides and diselenides. 

Herein, we review an anticancer and a chemopreventive activity of both selenides and diselenides, with a special attention to mechanisms of their biological action. The question of diselenides symmetry, as a feature favorable for anticancer activity [38], is also under consideration.

## 2. Selenides and Diselenides with Therapeutic Perspective

### 2.1. Naturally Occurring Selenides and Diselenides

Diselenides have received significant attention, not only because this moiety is usually found among the metabolites of the dietary compounds but also particularly because they have been postulated as one of the most important species in redox-cycling. In line with this, selenocystine, a naturally occurring diselenide (**3**, Figure 1b), exhibits strong antioxidant properties [39] and has been considered in several studies related to chemoprevention. Thus, it was effective against 4-(methylnitrosamino)-1-(3-pyridyl)-1-butanone (NNK) induced lung tumors in a specific mouse model sensitive to chemical carcinogenesis (the A/J mouse model) when administered in a pre-initiation period to tumor development, or, alternatively, when supplementation was restricted to a post-initiation period [40]. 

In a different study, **3** was found to trigger apoptosis in human breast adenocarcinoma MCF-7 cells through kinase regulation [41]. Induction of apoptosis was also attributed to **3** in HepG2 human hepatoma cells and in isolated rat liver mitochondria [42]. In this case, the proposed mechanism of apoptosis involved the opening of the mitochondrial permeability transition pores (and the associated loss of *Δψ_m_*, mitochondrial swelling and cytochrome c release), which is mediated by the cross-linking of protein thiol groups and by the generation of superoxide radical anions (O_2_^•−^) through reaction with glutathione (GSH) [42]. Selenocystamine (**4**) also oxidized the protein thiol groups in isolated mitochondria and in crude extracts of HepG2 cells, but not in intact HepG2 cells. These lines of evidence suggest that the action was similar to that exerted by **3**, precluding its inability to cross the cell membrane. Additionally, dimethyldiselenide (**5**), another naturally occurring diselenide, has been reported to be active against oxidative damage [43]. 

### 2.2. Synthetic Diselenides

In addition to biologically existing diselenides, a variety of synthetic diselenides has exhibited protective potential. Due to the high number of diselenides, they are reviewed herein in function of their biological activity: antioxidant activity, prooxidant/redox-modulating, kinase-modulating, antiproliferative/cytotoxic and apoptotic. The chemical names of mentioned diselenides are summarized in Table 1. 

#### 2.2.1. Antioxidant Activities of Synthetic Diselenides: GPx-Like, Metal-Binding, Chemopreventive and Free Radical Scavenging Potential

**Figure 2:**
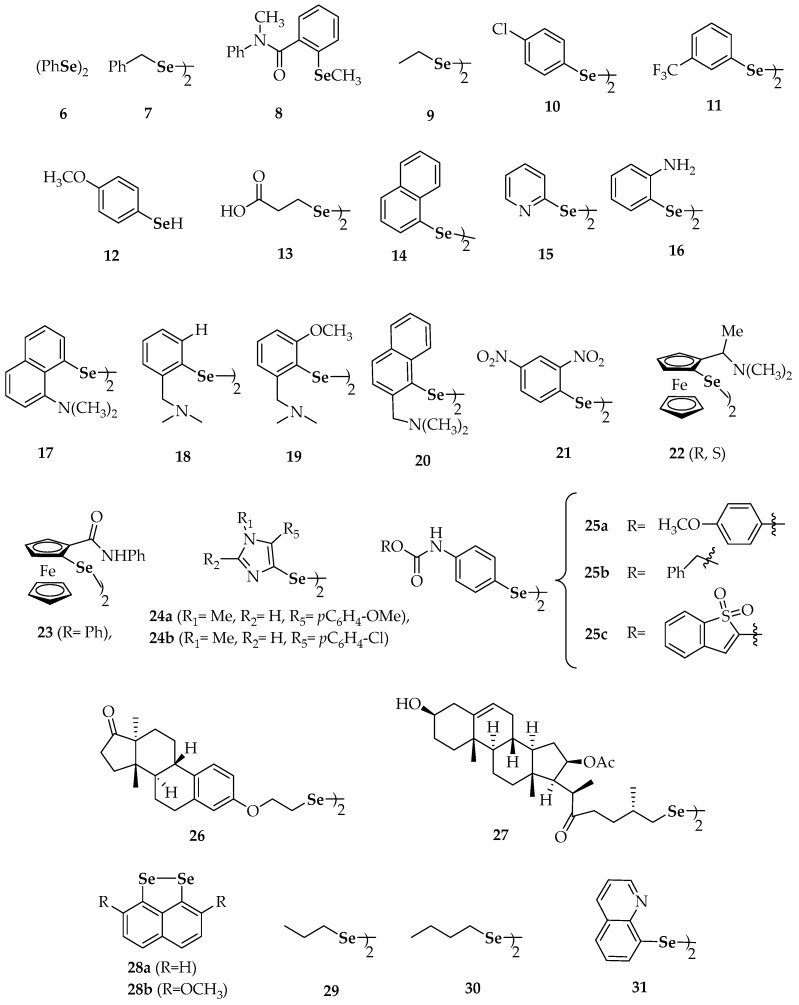
Synthetic diselenides and related selenides or selenols with antioxidant and/or prooxidant studies [44,45,46,47,48,49,50,51,52,53,54,55,56,57,58,59,60,61,62,63,64,65,66,67,68].

Diphenyl diselenide (**6**, Figure 2) has been evaluated in several studies and it is considered a reference compound in the evaluation of the antioxidant properties and of the glutathione peroxidase-like (GPx-like) activity [44,45,46,47,48]. Especially, **6** displayed a higher antioxidant activity than both dibenzyl diselenide (**7**) and a group of asymmetric selenides represented by **8** [44]. Concerning the protective role of diselenides on lipid peroxidation, **6** behaved as the most antioxidant compound among the different diaryl and dialkyl diselenides tested, regardless of the species (mice and rats) [46]. Dialkyl selenides, e.g., diethyl (**9**) and substituted diaryl diselenides such as di(*p*-clorophenyl) diselenide (**10**), were effective against lipid peroxidation but showed differential antioxidant potential in mice and rats, being more potent in mice [46].

The ability of diphenyl diselenide (**6**) to protect murine J774 macrophage-like cells from reactive oxygen species (ROS) generated by the oxidation of low density lipoproteins (LDL) was proved by Straliotto et al. [49]. The redox signaling effects of **6** were accompanied by the down-regulation of the nuclear factor-κB (NF-κB), a transcription factor involved in pro-apoptotic pathways activated by ROS. Melo et al. found that **6** was also able to protect breast (MCF-7) cancer cells from oxidative damage induced by tamoxifen hormone therapy without interfering with its cytotoxicity [50]. They concluded that, if used at low concentrations, **6** could be employed as a potent antigenotoxic agent to prevent the risk of cancer induction. 

The *meta*-CF_3_ substituted diphenyl diselenide (**11**) has been reported as an anti-mutagenic and anti-genotoxic selenium derivative [51]. It protected Chinese hamster lung fibroblasts (V79 cells) against H_2_O_2_-induced DNA damage at doses as low as 12.5 µM. In addition, **11** mimics catalase activity better than **6**, both compounds giving rise to different products upon reaction with H_2_O_2_.

The dimethyl- (**5**), diphenyl- (**6**) and dibenzyl diselenides (**7**) induced quinone reductase and glutathione-S-transferase activity [52]. The generated selenol derivatives (i.e., selenolates at physiological pH) can be considered as the metabolites responsible for such responses. 

Selenolates can be easily generated from selenols due to the high acidity of the Se-H bond (which is more labile than the equivalent S-H and O-H bonds because of the higher polarizability of selenium), that enables the rapid and almost complete ionization at physiological pH [53]. One of the first selenols assayed for inhibition of carcinogenesis has been *p*-methoxyphenylselenol (**12**), which showed effectiveness in the inhibition of azoxymethane-induced liver [54], colon and kidney [55] tumors in rats.

On the other hand, the antioxidant and GPx activity of 3,3′-diselenodipropionic acid (**13**) has been evaluated by Kunwar et al. [56]. The treatment with **13** prevented the depletion of the levels of endogenous antioxidants (GSH) in red blood cells through free-radical-induced stress. However, in comparison to sodium selenite or ebselen, its GPx activity was lower. Compound **13** has been also evaluated as a radio-protective agent against whole-body γ-radiation on Swiss albino mice [57]. In this in vivo study, authors concluded that **13** can attenuate radiation-induced DNA damage. Furthermore, it can inhibit radiation-induced apoptosis in the spleen and can reverse radiation-induced alterations in the expression of the pro-apoptotic BAX and the anti-apoptotic Bcl-2 genes.

Adding a second phenyl group to diphenyl selenide, converting it in binaphthyl diselenide (**14**), revealed that it showed a significant antioxidant activity [58]. Compound **14** could exert a protective action against 2-nitropropane (2-NP)-induced hepatoxicity in rats. 

Including nitrogen into the phenyl ring, converting it in a pyridine ring (**15**), has provided cytoprotective effects [59]. The 2-pyridyl derivative **15** caused the largest shift in the Cu^+2^ reduction potential and was also the most promising in the metallothionein (MT) assay for the measurement of catalytic activity. Similarly, the *N*-exocyclic aniline derivative **16** exhibited a noteworthy activity in the antioxidant assays considered. Indeed, **16** behaved as a better antioxidant than ebselen in human skin fibroblast (FCP7) cell culture under UVA-induced stress, thus suggesting that **16** could depict a leading compound for the development of more effective [46] multi-functional antioxidants. According to the authors, these different activities (GPx-like and metal binding characteristics of their nitrogen and chalcogen groups) could explain the extraordinary protection exerted by these compounds against the damage caused by ROS. The role of selenium-metal coordination in DNA damage inhibition was reported to be essential in a parallel study [60]. 

The naphthylamine derivative **17** did not show GPx-like activity when this activity was evaluated for selected intramolecularly-coordinated diorganyl diselenides [45]. In contrast, compounds with phenyl or naphthyl rings substituted with a (dimethylamino)methyl group (**18**–**20**) were active and these observations were attributed to the presence of better electron-donating groups in close proximity to selenium [61]. Likewise, a good activity was found for **21**, with an electron-withdrawing group (a nitro group) in *ortho* position to selenium. The same authors had previously evaluated the thiol peroxidase-like activity of related diselenides based on ferrocene, such as **22** [62]. The introduction of the ferrocene moiety increased the activity, although the most important effect was suspected to be exerted through the efficacy of the intramolecular N^…^Se coordination, as **23**, with an amide group and, thus, a less coordinating nitrogen atom, was only superior to **6** and **17** (GPx-like activity: **22** > **20** > **21** >> **18** > **23** >> **6** > **17**). 

Bhabak and Mugesh [63] reported the enhancement of the antioxidant activity of diselenides compounds such as **18** by the introduction of a methoxy group as a substituent in *ortho* to selenium (**19**). The observed increment of activity in compound **19** was investigated through kinetic and computational methods and ascribed to the role of the methoxy group in altering the steric and electronic environment around selenium, in particular, preventing over-oxidation by peroxides and preventing thiol-exchange reactions in the selenenylsulfide intermediate of the catalytic cycle. The same authors also found an enhanced GPx activity of *sec*-amino-substituted diselenides analogous versus the analogous *tert*-amine-based compounds [64]. 

The *p*-methoxyphenyl- (**24a**) and *p*-chlorophenyl-substituted (**24b**) compounds were the most active molecules in a series of related imidazole-containing derivatives assayed for their GPx-like activity and their capacity to be reduced by glutathione (GSH) [65]. Regarding GPx-like activity, they were 4-fold more potent than ebselen, whilst they showed 3–5-fold more reactivity toward GSH than **6**. In general, the most effective compounds were those substituted at 5-position of the imidazole ring, close to the selenium atom. Apart from this fact, no other clear structure–activity relationship emerged from these antioxidant studies. 

An interesting study by Romano et al. [66] compared the capability of new selenocarbamate derivatives to scavenge the radical 1,1-diphenyl-2-picryl-hydrazyl (DPPH) and 2,2-azinobis(3-ethylbenzothiazoline-6-sulfonic acid) (ABTS), as well as their ability to mimic GPx activity. Two families containing common active fragments, consisting of selenocyanate and diselenide derivatives, did not differ much in their activities despite double amount of selenium (on a molar basis) and symmetry in the case of diselenides. Among diselenides, **25a**, **25b** and **25c** (Figure 2) displayed potential radical scavenging activity for DPPH and ABTS, although they did not exhibit GPx-like activity. 

When evaluating the GPx-like activity of a group of selenosteroids (**26** and **27**, among others), Fuentes-Aguilar et al. [67] found that the GPx-like activity was under that of ebselen for all the compounds considered.

On some occasions, the chemoprotective activity of diselenides has been related to conformational restriction. Thus, certain *peri*-diselenides (**28a** and **28b**) have shown superior GPx-like activity than acyclic diaryl diselenides [68]. It should be expected that several properties of the compounds worked synergistically, however, as the introduction of methoxy groups in the naphthalene moiety made chemoprevention increase for derivative **28b** in comparison to **28a**.

#### 2.2.2. Prooxidant Activity and Redox Modulation Activity of Synthetic Diselenides

Dialkyl diselenides (**29** and **30**), in contrast to diaryl ones (**6**, **10**, and **11**), proved to behave as prooxidants at low concentrations [46]. According to in vivo studies and despites its above-mentioned antioxidant potential, **6** can also be toxic in certain conditions, depending on dose, species and route of administration [69,70].

The intracellular redox modulation by selenocompounds has been deeply studied. Nevertheless, the extracellular redox control has been only recently linked to the cellular uptake and to the cytotoxicity of selected selenium-containing derivatives [71,72]. The extracellular redox status, in whose regulation both cell surface and secreted redox-active proteins are involved, seems to be correlated with several biological responses such as cell proliferation and apoptosis. The cysteine/cystine couple is attributed to play a key-role in the extracellular redox state, whereas thioredoxin (Trx), thioredoxin reductase (TrxR) and protein disulfide isomerase (PDI) are among the proteins whose expression may increase the production of cysteine. Apart from phenylselenol (PhSeH) and ebselen, diphenyl diselenide (**6**, Figure 2) and dibenzyl diselenide (**7**) have been pointed as inductors of extracellular cysteine accumulation in murine macrophage RAW 264.7 cells and in differentiated human THP-1 monocytes [72]. Nevertheless, neither selenocystine (**3**, Figure 1) nor methylselenocysteine could exert the same effects. The authors proposed a model where production of extracellular cysteine increases because of the induction of TrxR1 expression on the cell surface by certain organoselenium compounds. Again, selenolates are pointed as the putative bioactive species, as **6** and PhSeH showed nearly identical cellular effects on a molar basis [72]. 

Diethyl- (**9**) and dipropyl (**29**) diselenides demonstrated a higher potential for –SH group oxidation than diaryl diselenides, presumably because the generated selenolates would be more nucleophilic. Dibutyl diselenide (**30**) was prooxidant at low concentrations [46]. 

In contrast to endocyclic (**15**) and exocyclic (**16**) N-containing monocyclic diselenides, the diquinolyl-8-yl diselenide (**31**) containing fused-ring systems acted as a prooxidant at 5 µM, although it exerted a slightly protective action at 1 µM [59]. 

#### 2.2.3. Kinase Modulation Activity of Synthetic Diselenides

**Figure 3:**
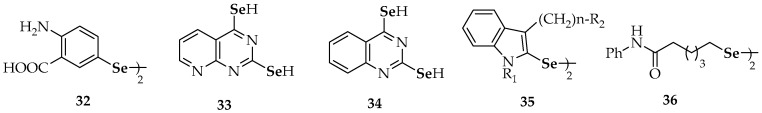
Synthetic diselenides and related selenols with kinase modulation properties [73,74,75,76].

In the frame of kinase modulation as a possible pathway in the mechanisms of action of selenocompounds as anticancer agents [73], a library of organoselenium compounds was analyzed by Plano et al. [74]. Compounds with a variety of aromatic, heteroaromatic and aliphatic nitrogen-containing moieties (**32**–**36**, Figure 3) showed a noteworthy influence over different kinases. Among them, the bis(4-amino-3-carboxyphenyl) diselenide derivative **32** showed a mild inhibition of cyclin kinase (CDKs) activity. On the other hand, selenol-disubstituted pyrido[2,3-d]pyrimidine (**33**) and quinazoline (**34**) derivatives were proposed as promising candidates against cancer and other diseases: they are capable to produce a potent activation of the insulin-like growth factor 1 receptor (IGF-1R), playing thus a major role in cancer growth, tumor cell survival and resistance to therapy [75]. In line with the former compounds, the indol-containing diselenide **35** acted through inhibition of tyrosine kinase [73].

The antitumoral effect of the amide-containing derivative **36** [76] has been partially attributed to its capability for inhibiting the PI3 kinase pathway (see Section 2.2.4). 

#### 2.2.4. Antiproliferative and Cytotoxic Activity of Synthetic Diselenides

There are several Se-based compounds that can inhibit the growth of malignant cells. The challenge of such compounds is selectivity, in the sense that the effect on normal cells should ideally be marginal. Among the compounds with antiproliferative potential, **5** (Figure 1), **6** and **7** (Figure 2), which have been already mentioned with regard to their antioxidant activity, inhibited cell growth in murine hepatoma (Hepa 1c1c7) cells [52]. The authors pointed out that the inhibition of cell growth correlated with their potential to induce quinone reductase and glutathione-S-transferase activity. By contrast, the 3,3′-diselenodipropionic acid **13**, described as a radio-protective agent and a free-radical scavenger, was not found to be toxic toward lymphocytes and EL4 tumor cells at the concentrations used [56]. 

Regarding the phenylcarbamate diselenides **25a**, **25b** and **25c** (Figure 2) [66], which showed radical scavenging activity but lacked in GPx activity (mentioned above), they displayed the best in vitro antiproliferative activities among several tested diselenides against a panel of human tumor cell lines including lung carcinoma (HTB-54), colon carcinoma (HT-29), lymphocytic leukemia (CCRF-CEM), breast adenocarcinoma (MCF-7) and prostate adenocarcinoma (PC-3). Unfortunately, most compounds exhibited similar activities in both tumor and non-tumor cell lines. By a subsequent molecular modeling analysis [77], it was concluded that the cytotoxic activity correlated with the greater or lesser ability to release the active fragments: the central common fragment, the alcohol or phenols moieties, or the corresponding selenol (RSeH) and selenenic acid (RSeOH) derivatives. 

For estrone and diosgenin-based diselenides (**26** and **27**)**,** the in vitro antiproliferative activity against a panel of six human solid tumor cell lines, when tested up to a 100 µM concentration, was negligible [67]. Cancer cell lines included non-small cell lung (A549 and SW1573), breast (HBL-100 and T-47D), cervix (HeLa) and colon (WiDr) [67]. 

**Figure 4:**
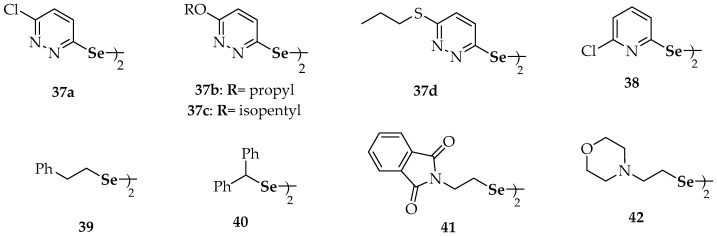
Synthetic diselenides with antiproliferative studies [52,56,66,67,76,77,78,79].

Substituted dipyridazinyl diselenides **37a**–**d** (Figure 4), substituted diaralkyl diselenides (**7**), substituted dipyridinyl diselenides **15** and **38**, di(phenylethyl) diselenide (**39**), dibenzhydryl diselenide (**40**), the phthalimide-containing diselenide **41** and the morpholine-containing diselenide **42** (Figure 2 and Figure 4, respectively) have been recently evaluated on their antiproliferative ability against human breast cancer MCF-7 cells [78]. The chloro-substituted pyridazine derivative **37a** showed the highest potency, markedly inhibiting MCF-7 cell growth with an IC_50_ value of 10.34 µM, in a dose-dependent manner. In addition, other seven diselenides (**37b**, **37c**, **37d**, **38**, **15**, **41** and **42**) showed higher potency, in terms of IC_50_, than the positive control (5-fluorouracil) against MCF-7 cells, suggesting the potential anticancer activity of these selenocompounds. The relatively broad diversity of structures that gave rise to antiproliferative activities makes it difficult to deduce a structure–activity relationship valid to explain the results, apart from the consideration of the symmetry that implies the presence of the diselenide moiety in all of these compounds [78]. 

Two of the active compounds in the abovementioned study of Kim et al. [78], phthalimide (**41**) and morpholine (**42**) derivatives, depicted sp^3^ carbon atoms directly bonded to selenium. As indicated above, most part of synthetic selenide-derivatives with reported anticancer activity depict Csp^2^-Se bonds, even though methylselenol has been proposed as the critical selenium metabolite of the dietary seleno-amino acids for anticancer activity [79]. Nevertheless, also compound **36** (Figure 3), described by Gowda et al. [76], has alkylic C-Se bonds. A promising anticancer activity has been reported for this symmetrical diarylalkyl diselenide. Compound **36** was tested in the prevention of early melanocytic lesion development in laboratory-generated skin reconstructs of WM35 or WM115 melanoma cell lines [76]. The topical application of **36** inhibited melanoma by up to 87% with negligible toxicological effects. Mechanistically, its action corresponds to a histone deacetylase (HDAC) and PI3 kinase pathway inhibitor. This combination of actions explains its efficacy in comparison to suberoylanilide hydroxamic acid (SAHA), the best-known HDAC inhibitor at the time. Furthermore, results suggested the efficacy of the indole derivative **35** (Figure 3) against other cancer types, as it was also able to inhibit cell growth in pancreatic (MIA-PA-Ca-2), breast (MDA-MB-231), prostate (PC-3) or sarcoma (HT-1080) cell lines.

#### 2.2.5. Apoptotic Activity of Synthetic Diselenides

**Figure 5:**
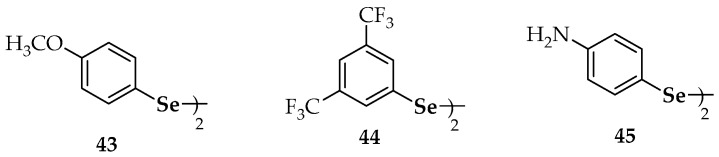
Synthetic diselenides with apoptotic activity [80,81,82].

Diphenyl diselenide (**6**) can down-regulate the expression of the nuclear factor-κB (NF-κB), which is a transcription factor involved in pro-apoptotic pathways activated by ROS [80]. Similarly, substituted diaryl diselenides **11** (Figure 2) and **43** (Figure 5) were shown to induce apoptosis in human colon adenocarcinoma cells (HT-29) [80]. Nedel et al. used the 3-(4,5-dimethylthiazol-2-yl)-2,5-diphenyl-tetrazolium bromide (MTT) assay and flow cytometry analyses to show the effect of **11** and **43**. They proposed that such action was produced through activation of caspase-dependent and independent pathways, in addition to cell-cycle arrest mediated by the p53, p21 and MYC genes.

The cytotoxic and apoptotic potential of diselenide **44** (Figure 5) has been confirmed though DNA cell cycle analysis and through microscopic monitoring of the formation of apoptotic bodies [81]. Among the 13 symmetric aromatic diselenides with a variety of substitution patterns evaluated by Rizvi et al. [81], compound **44** showed the highest inhibition of cell growth in human leukemia HL-60 cells (IC_50_ = 8 µM). Its cytotoxic potential against prostate (PC-3), breast (MCF-7), pancreatic (MIA-PA-Ca-2) and colon (HCT-116) cancer cell lines was also demonstrated (IC_50_ values of 13, 18, 25 and 27 µM, respectively). The antiproliferative activity of **44** on HL-60 cells involved the inhibition of the S phase of the cell cycle and the induction of apoptosis through the mitochondrial-dependent pathway. The authors also determined the DNA binding ability of **44** using molecular docking, showing that binding of the compound occurs selectively to the minor groove of DNA through hydrogen bonds involving the fluorine atoms. This binding was reinforced through hydrophobic interactions mediated by the benzene rings. Interestingly, pyridine-containing compound **15** (Figure 2) did not show any remarkable pro-apoptotic potential against the series of cell lines mentioned above [81].

A comprehensive library including nine symmetrical diarylalkyl diselenides (Ar-(CH_2_)*_n_*-Se-Se-(CH_2_)*_n_*-Ar; *n* = 0, 1) has been assayed for apoptotic potential in several cell lines [82]. Breast adenocarcinoma (MCF-7) cells were considered, as well as human prostate (PC-3) cells, lymphocytic leukemia (CCRF-CEM) cells and human colorectal adenocarcinoma (HT-29) cells. Again, it was a diaryl diselenide (**45**, Figure 5) the compound which displayed the best therapeutic profile regarding superoxide generation and cell cytotoxicity. Thus, aniline derivative (**45**) was the most potent organoselenium compound (among 59 candidates) against human prostate (PC-3) cells (IC_50_ = 1.7 µM) and was 8-fold more active than etoposide (IC_50_ = 13.6 µM), an agent commonly used in the treatment of prostate cancer. Compound **45** also exhibited more cytotoxic potency (IC_50_ = 4.3 µM) than etoposide (IC_50_ = 17.5 µM) in MCF-7 cells and showed noteworthy cytotoxic effects both in CCRF-CEM cells (IC_50_ = 9.0 µM) and HT-29 cells (IC_50_ = 9.8 µM). Additionally, compound **45** affected the cell cycle distribution of MCF-7 cells in the G2/M and S-phases. Moreover, **45** developed an apoptotic activity in CCRF-CEM cells, mediated by reactive oxygen species (ROS) and similar to camptothecin, the pro-apoptotic compound used as positive control.

### 2.3. Synthetic Selenides

Organic selenides are less reported in the literature of anticancer reagents in comparison to diselenide derivatives. Herein, the studies involving selenides are divided in two main activities: antioxidant (redox modulating, antioxidant and chemopreventive compounds) and antitumoral compounds (derivatives with antiproliferative, cytotoxic and apoptotic activity). The chemical names of the mentioned selenides are summarized in Table 2.

#### 2.3.1. Redox-Modulating and Antioxidant and Chemo-Preventive Activities of Synthetic Selenides

The diallyl selenide **46** (Figure 5) exhibited significant anti-carcinogenic activity when assayed in a murine (DMBA)-induced mammary tumor model in a study performed to compare the chemopreventive potential of organoselenium compounds versus the corresponding sulfur analogs against cancer [83]. Through administration in a pre-initiation period to tumor development, the protective activity of this symmetric selenide resulted around 300 times superior to its sulfur isoster (diallyl sulfide). In a more recent research work by Mecklenburg et al. [84], the piperazine compound **47**, which combines redox catalysis with metal binding properties, was virtually non-toxic (at concentrations up to 50 µM) against human SK-Mel-5 melanoma cells in the absence of H_2_O_2_ (91% survival at 25 µM), whereas it showed a toxicity in the presence of H_2_O_2_ 2–3-fold higher than in its absence (65% survival at 25 µM). As compound **47** (Figure 5) was somehow able to recognize the particular redox state of the cells and, according to this state, exert either an antioxidative or a prooxidative action, it has been considered a sensor/effector agent.

Some additional selenides have been studied from the point of view of their antioxidant activity, but on these occasions, they have not been explicitly connected to anticancer research. For instance, Kumakura et al. [85] evaluated the GPx-like activity [86] of the water-soluble cyclic selenide **48** (Figure 5). In the same way as it occurred with cyclic diselenides **28** (Figure 2), the derivative **48** exerted higher antioxidant catalytic activity than its linear analogs **49**. According to the authors, conformational restriction increased the HOMO energy level and made the selenium atom more exposed. Other selenides, such as **50a**–**c** (Figure 5), provided fast reaction rates for the reduction of H_2_O_2_ with thiols by both high-performance liquid chromatography (HPLC) and nuclear magnetic resonance (NMR)-based assays [87]. The fact that rates were more elevated than for their unsubstituted or otherwise methoxy-substituted analogues was attributed to resonance stabilization of increased positive charge on the selenium atom during the rate determining oxidation step. The polyethylene glycol (PEG) derivatives **50d** and **50e** were prepared to increase aqueous solubility and they also afforded remarkable activity. Noteworthy, both the non-PEGylated and PEGylated compounds that revealed the best activities gave rise spirodioxyselenuranes adducts upon oxidation, while derivatives with diminished activity generated pincer compounds. 

Prevention of oxidative damage and radical scavenging potential were found for the indol-selenide derivative **51 [88]** and the 6-arylselanylpurines **52a** and **52b** [89], respectively. Apart from preventing oxidative stress, compound **51** did not show hepatic, renal or cerebral toxicity. Regarding derivatives **52a** and **52b**, both presented a significant ABTS radical scavenging activity at concentrations equal/higher than 100 µM, although only the latter displayed moderated DPPH radical scavenging activity, at a concentration of 200 µM.

The multifunctional redox naphthalene-1,4-dione selenides (**53**, **54a**, **54b** and **55**), which combine more than one redox site in one molecule including a quinone moiety, are particularly suitable to target (cancer) cells under ROS. Compound **53** seemed to trigger the formation of superoxide in the mitochondria, since a yeast mutant deficient in superoxide dismutase 2 (SOD2) was hypersusceptible to it [90]. On the other hand, compound **54b** exhibited similar GPx-like activity to ebselen [91], increasing the initial rate of the reaction by 1.95-fold (ebselen increased the rate by 1.87-fold). 

The in vitro antioxidant activities of several chalcogen-based zidovudine (3′-Azido-3′-deoxythymidine, AZT) analogues have been explored [92]. Firstly, the thiobarbituric acid reactive substance (TBARS) assay allowed the quantification of the final products of lipid peroxidation in brain homogenates and showed excellent results for compounds **56b** and **56c** at two different concentrations (100 and 200 µM). Additionally, the same compounds were able to decompose H_2_O_2_ efficiently and this decomposition involved a 4–5-fold decrease in times required to oxidize 50% of the benzenethiol in comparison to DMSO (control) and to therapeutically employed AZT. 

#### 2.3.2. Antitumoral Activity of Synthetic Selenides

**Figure 6:**
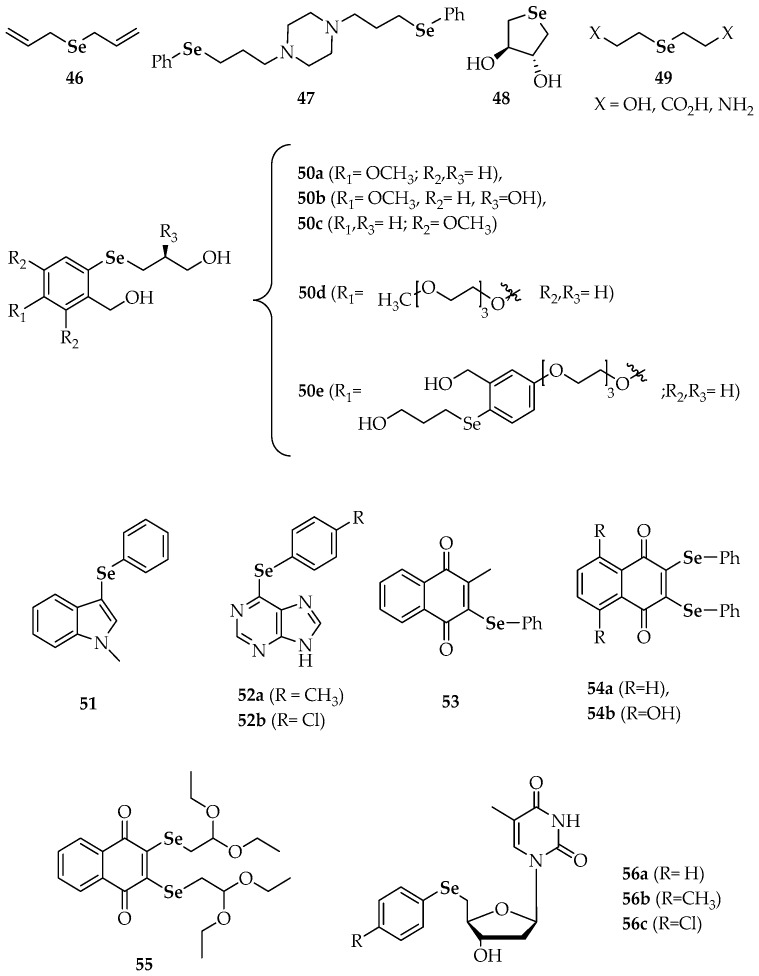
Synthetic selenides with antioxidant studies [83,84,85,86,87,88,89,90,91,92].

To gain an initial overview of possible cytotoxicity of the naphthalene-1,4-dione derivatives (**53**, **54a**, **54b** and **55**, Figure 6), an activity screening was performed in human myeloid leukemia K562 cells [91]. Compound **55** was found to be particularly active at 10 µM, with an average remaining cell viability of just 8.6%. However, the effect of the latter might also be related to the protected aldehyde (acetal) function: the reactive aldehyde could be liberated in cells and exert its own toxic effects. When tested in chronic lymphocytic leukemia (CLL) B-cells isolated from the peripheral blood (PB) of leukemia patients, compounds **54a** and **54b** showed strong activity in reducing CLL B-cell viability at low to submicromolar concentrations [91]. At the same concentrations, viability of healthy donor peripheral blood mononuclear cells (PBMC) was unaffected by **54a** whereas a high toxicity to healthy control cells was observed for compound **54b**. Thus, compound **54a** was the most effective in selectively reducing the viability of CLL cells. This effect was particularly pronounced at 500 nM and 1 µM. Fludarabine, the major single chemotherapeutic component used in CLL at the moment, only afforded around 30% reduction of CLL B-cell survival in vitro when used at clinically feasible doses. The significant reduction of cell survival achieved by **54a** at concentrations as low as 500 nM means that it compares well with such established cytostatics, at least in vitro.

**Figure 7:**
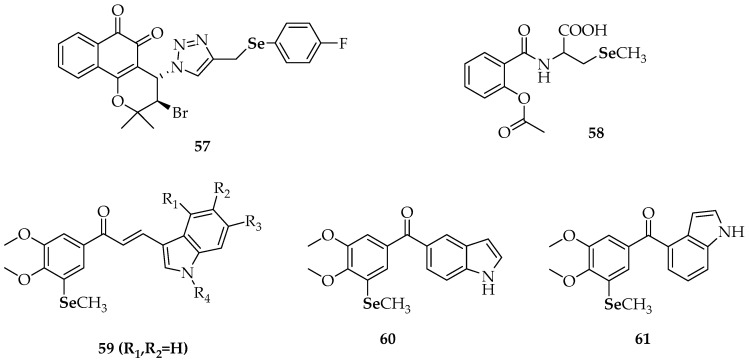
Synthetic selenides with antitumoral studies [91,92,93,94,95].

In relation to the above mentioned quinone-containing selenides, the antiproliferative activity of the 1,2-naphthoquinone-containing selenide (**57**, Figure 7) was found to be superior to the activity of other 1,2- and 1,4-naphthoquinone-based chalcogen derivatives [93]. While the IC_50_ values of the latter were between 10 and 100 µM and for other 1,2-naphthoquinone derivatives were between 1 and 10 µM, compound **57** was active even in sub-micromolar concentrations against human promyelocytic leukemia cells (HL-60) and human colon carcinoma cells (HCT-116). The remarkable activity of **57** was attributed to three factors: the 1,2-naphthoquinone moiety, the Se atom and the electron-withdrawing substituent in *para*-position to Se. Unfortunately, no selective cytotoxicity against cancer versus normal cells was detected. 

One of the major limitations in the clinical use of AZT is associated with hepatotoxicity. In fact, AZT caused a significant reduction in hepatic cell viability at concentrations of 200, 100 and 50 µM compared with the above-mentioned compound **56c** (Figure 6) at 200 µM [92]. When the cytotoxicity against bladder carcinoma cells (5637) was tested, AZT proved to be more effective than its derivatives in inhibiting tumor growth at the lowest concentrations. Nevertheless, at the highest concentrations, **56a**–**c** were more effective and did not cause cellular injury. Derivatives **56a**–**c** also showed better pro-apoptotic properties than AZT in 5637 cells according to annexin V-binding assays. Thus, at 50 µM, **56c** induced around 70% of apoptosis whereas AZT caused around 8% of apoptosis. Additionally, the percentage of apoptosis reached 90% by exposure of 5637 cells to **56a**–**c** at 100 µM. The analysis of DNA fragmentation revealed that **56a**–**c** significantly increased apoptosis-associated DNA fragmentation comparing to AZT. When the gene expression of anti-apoptotic Bcl-2 and pro-apoptotic BAX was investigated, the authors found that the BAX: Bcl-2 ratio was affected by these compounds. Whereas Bcl-2 mRNA levels were significantly lower (compared to control) in cells exposed to both AZT and **56a**–**c**, BAX mRNA levels were significantly higher except for cells exposed to AZT. Moreover, a predominance of BAX to Bcl-2 levels, as well as significant increases in caspase-9 mRNA levels, were observed for compounds **56a** and **56b** in comparison with the controls.

Plano et al. [94] evaluated the anticancer activity of several synthetic anti-inflammatory drug-hybrid molecules against four cancer cell lines. Among the compounds tested, the derivative **58** (Figure 7), which is a conjugate of selenocysteine with aspirin, was only moderately cytotoxic to human pancreatic carcinoma cell line (PANC-1 cells). The most active compound was an aspirin analogue N-substituted with a 2-selenocyanoethyl group, which was particularly effective in reducing the viability of colorectal cancer (CRC) cells.

Very recently, Zhang et al. [95] studied the antiproliferative activity of several synthetic indole-chalcone derivatives **59** and the phenylindolyl ketone derivatives **60** and **61** (Figure 7). Most of these compounds exhibited good to excellent activity (with IC_50_ values in the sub-micromolar level) in six human cancer cell lines: non-small-cell-lung cancer cells (A549), human breast cancer cells (MDAMB-231), human liver carcinoma cells (HepG2), human epithelial cervical cancer cells (HeLa), human colon carcinoma cells (HCT-116 and RKO). Among the indole-chalcone derivatives, the non-substituted indole-3-carbaldehyde **59** exhibited the highest activity (23 to 79 nM). The authors found that an *N*-methyl group in the indole moiety was unfavorable and that the activity was related to the position of substitution on the indole rings. Compound **61** (Figure 7), a phenylindolyl ketone derivative, exhibited the most potent antiproliferative activities against the six human cancer cell lines with IC_50_ values between 4 nM for the A549 cell line and 22 nM for RKO cells. A microtubule dynamics assay and an immunofluorescence assay confirmed that **61** could effectively inhibit tubulin polymerization in HeLa cells (IC_50_ = 2.1 ± 0.27 µM). Further cellular mechanism studies revealed that **61** induced G2/M phase arrest and that apoptosis was associated with a decrease in the mitochondrial membrane potential *Δψ_m_*. Compared to the reference compound showing an oxygen atom instead a Se atom, (1*H*-indol-4-yl)(3,4,5-trimethoxyphenyl)methanone, a remarkable effect of substitution by selenium was observed, suggesting that the introduction of selenium was beneficial to the antitumor activity. The activity of **61** also pointed out that the joint position of benzoyl on the indole ring was important. 

## 3. Conclusions

Our overview of current selenium compounds with anticancer and chemopreventive properties indicates the predominant role for the diaryldiselenide family that is also wider investigated than both selenides and dialkyldiselenides. 

An analysis of the comprehensive studies on (di)selenide compounds shows some structure–activity relationship that can be useful for further search of new selenium anticancer agents with therapeutic perspective. Thus, the most potent anticancer properties, with the widest spectrum of protein targets and mechanisms involved in their action, can be observed for symmetric diselenides with direct aromatic moieties. The alkyl spacer between an aryl ring and Se usually causes a decrease of the desirable biological actions. 

The role of the aromatic ring properties, including their size, presence of heteroatoms as well as the number and kind of substituents, is crucial for potency and decisive for mechanisms of anticancer/chemopreventive action. Various lines of evidence underline an importance of nitrogen atom, occurred either as exo-amine substituents or as endocyclic heteroatoms of pyridine, pyridazine, quinolines, indol or purines. More or less electron-withdrawing substituents, i.e., halogens, trifluoromethyl or nitro groups, at the diphenyl diselenides aromatic rings, play also an important role and can be found within the structures of apoptosis agents of particularly dangerous human cancer lines. However, unsubstituted aromatic rings of diphenyl diselenides have been confirmed as beneficial for various mechanisms of anticancer and chemopreventive activities, including protection of murine J774 macrophage-like cells from ROS, an action involving down-regulation of NF-κB or anti-genotoxic one. 

On the other hand, some studies confirm a favorable effect of the electro-donating methoxy-substituent that causes the chemoprevention increase. The extensive results of anti-cancer properties of a significant number of selenocompounds show that their symmetric topology is of particular importance. This is observed not only for diaryl, but also for dialkyl diselenides and for the less extended family of the selenide agents. Among selenides, the most potent anticancer properties were identified for the symmetric diallylselenide, although the cyclic compounds are confirmed as more potent than linear ones. The recent search for anticancer agents has also provided some data for niche groups of selenium compounds, i.e., the active ferrocene- and peri-diselenides and the less active selenosteroids. 

Considering both the potent anticancer/chemopreventive activity and various mechanisms of the biological action, selenides and, especially, diselenides described to date, create a library of “small molecule catalysts” that can be useful for further stages of the antitumor drug research and development process to give a high probability of finding new therapies against cancer.

## Figures and Tables

**Table 1:** Synthetic diselenides mentioned.

Compound			Compound
**6**	diphenyl diselenide	**26**	bis[2-(estra-1,3,5(10)-trien-1 7-one-3-yl)oxyethyl]diselenide
**7**	dibenzyl diselenide	**27**	bis[(25*R*)-16β-acetoxy-3β-hydroxy-22-oxocholest-5-en-26-yl]diselenide
**9**	diethyl diselenide,	**28a**	naphtho[1,8-cd][1,2]diselenole
**10**	bis(4-clorophenyl) diselenide	**28b**	3,8-dimethoxynaphtho[1,8-cd][1,2]diselenole
**11**	3′3-ditrifluoromethyldiphenyl diselenide	**29**	dipropyl diselenide
**12**	*p*-methoxybenzeneselenol	**30**	dibutyl diselenide
**13**	3,3′-diselenodipropionic acid	**31**	diquinolyl-8-yl diselenide
**14**	binaphthyl diselenide	**32**	4,4′-diamino-3′,3-dicarboxydiphenyl diselenide
**15**	dipyrid-2-yl diselenide	**36**	6,6′-diselanediylbis(*N*-phenylhexanamide)
**16**	2′2-diaminodiphenyl diselenide	**37a**	6,6′-dichlorodipyridazyn-2-yl diselenide
**17**	8-(2-(8-(dimethylamino)naphthalen-1-yl)diselanyl)-*N*,*N*-dimethylnaphthalen-1-amine	**37b**	6,6′-di(propoxy)dipyridazyn-2-yl diselenide
**18**	(2-(2-(2-((dimethylamino)methyl)phenyl)diselanyl) phenyl)-*N*,*N*-dimethylmethanamine	**37c**	6,6′-di(isopentyloxy)dipyridazyn-2-yl diselenide
**19**	2-(2-(2-((dimethylamino)methyl)-6-methoxyphenyl) diselanyl)-3-methoxyphenyl)-*N*,*N*-dimethylmethanamine	**37d**	6,6′-di(propylthio)dipyridazyn-2-yl diselenide
**20**	(1-(2-(2-((dimethylamino)methyl)naphthalen-1-yl)diselanyl)naphthalen-2-yl)-*N*,*N*-dimethylmethanamine	**38**	2-chloro-6-(2-(6-chloropyridin-2-yl)diselanyl)pyridine
**21**	1,2-bis(2,4-dinitrophenyl)diselane	**39**	2,2′- di(phenylethyl) diselenide
**22**	bis(2-(1(*R*,*S*)-(*N*,*N*-dimethylamino)ethyl)ferrocen-1-yl) diselenide	**40**	dibenzhydryl diselenide
**23**	bis(2-(*N*-phenylcarbamoyl)ferrocen-1-yl) diselenide	**41**	1,2-bis(isoindoline-1,3-dione-2 ethyl)diselane
**24a**	5-(4-methoxyphenyl)-4-(2-(5-(4-methoxyphenyl)-1-methyl-1*H*-imidazol-4-yl)diselanyl)-1-methyl-1*H*-imidazole	**42**	1,2-bis(2-morpholinoethyl)diselane
**24b**	5-(4-chlorophenyl)-4-(2-(5-(4-chlorophenyl)-1-methyl-1*H*-imidazol-4-yl)diselanyl)-1-methyl-1*H*-imidazole	**43**	4′,4-dimethoxydiphenyl diselenide
**25a**	bis-4-[1-[(4′-methoxy)phenyl]-4-seleno-imidazole]	**44**	3′,5′,3,5-tetratrifluoromethyl-diphenyl diselenide
**25b**	4,4′-diselanediylbis(*N*-benzylbenzamide)	**45**	4′,4-diaminodiphenyl diselenide
**25c**	*N*-(1,1-dioxidobenzo[b]thiophen-2-yl)-4-((4-((1,1-dioxidobenzo[b]thiophen-2-yl)carbamoyl)phenyl) diselaneyl)benzamide		

**Table 2:** Selenides mentioned in the text.

Compound	
**46**	diallyl selenide
**47**	1,4-bis(3-(phenylselanyl)propyl)piperazine
**48**	*trans*-3,4-dihydroxyselenolane
**50 a*–*e**	different substituted phenyl 3-hydroxypropyl selenides
**51**	3-(phenylselanyl)-1-methyl-1*H*-indole
**52a**, **52b**	6-(4-methylphenyl)selanylpurine 6-(4-chlorophenyl)selanylpurine
**53**	2-methyl-3-(phenylselanyl) naphthalene-1,4-dione
**54a**	2,3-bis(phenylselanyl)naphthalene-1,4-dione
**54b**	5,8-dihydroxy-2,3-bis(phenyl-selanyl)naphthalene-1,4-dione
**55**	2,3-bis((2,2-diethoxyethyl)selanyl)naphthalene-1,4-dione
**56a**	5′-(phenylseleno)zidovudine
**56b**	5′-(4-methylphenylseleno)zidovudine
**56c**	5′-(4-chlorophenylseleno)zidovudine
**57**	3-bromo-4-(4-(((4-fluorophenyl)selanyl)methyl)-1*H*-1,2,3-triazol-1-yl)-2,2-dimethyl-3,4-dihydro-2*H*-benzo[h]chromene-5,6-dione
**58**	2-(2-acetoxybenzamido)-3-(methylselanyl)propanoic acid
**59**	selenide-containing indole chalcone derivatives
**60**	(3,4-dimethoxy-5-(methylselanyl)phenyl)(1*H*-indol-5-yl)methanone
**61**	(3,4-dimethoxy-5-(methylselanyl)phenyl)(1*H*-indol-4-yl)methanone

## Abbreviations

NNK	4-(methylnitrosamino)-1-(3-pyridyl)-1-butanone
GSH	Reduced glutathione
GPx-like	Glutathione peroxidase-like
ROS	Reactive oxygen species
LDL	Low density lipoproteins
NF-κB	Nuclear factor-κB DNA Binding
MTT	3-(4,5-dimethylthiazol-2-yl)-2,5-diphenyl-tetrazolium bromide
Trx	Thioredoxin
TrxR	Thioredoxin reductase
PDI	Disulfide isomerase
2-NP	2-Nitropropane
MT	Metallothionein
CDKs	Cyclin kinases
IGF-1R	Insulin-like growth factor 1 receptor
HDAC	Histone deacetylase
SAHA	Suberoylanilide hydroxamic acid
DPPH	1,1-Diphenyl-2-picryl-hydrazyl
ABTS	2,2-Azinobis(3-ethylbenzothiazoline- 6-sulfonic acid)
DMBA	7, 12-dimethylbenz(*a*)anthracene
HPLC	High-performance liquid chromatography
NMR	Nuclear magnetic resonance
PEG	Polyethylene glycol
SOD2	superoxide dismutase 2
PB	peripheral blood
PBMC	peripheral blood mononuclear cells
